# Advancing Brain-Computer Interface Applications for Severely Disabled Children Through a Multidisciplinary National Network: Summary of the Inaugural Pediatric BCI Canada Meeting

**DOI:** 10.3389/fnhum.2020.593883

**Published:** 2020-12-03

**Authors:** Eli Kinney-Lang, Dion Kelly, Erica D. Floreani, Zeanna Jadavji, Danette Rowley, Ephrem Takele Zewdie, Javad R. Anaraki, Hosein Bahari, Kim Beckers, Karen Castelane, Lindsey Crawford, Sarah House, Chelsea A. Rauh, Amber Michaud, Matheus Mussi, Jessica Silver, Corinne Tuck, Kim Adams, John Andersen, Tom Chau, Adam Kirton

**Affiliations:** ^1^Department of Pediatrics and Clinical Neurosciences, Cumming School of Medicine, University of Calgary, Calgary, AB, Canada; ^2^Cumming School of Medicine, Alberta Children’s Hospital Research Institute, University of Calgary, Calgary, AB, Canada; ^3^Cumming School of Medicine, Hotchkiss Brain Institute, University of Calgary, Calgary, AB, Canada; ^4^Department of Rehabilitation Science, Institute of Biomedical Engineering, University of Toronto, Toronto, ON, Canada; ^5^PRISM Laboratory, Bloorview Research Institute, Holland Bloorview Kids Rehabilitation Hospital, Toronto, ON, Canada; ^6^I CAN Centre, Glenrose Rehabilitation Hospital, Alberta Health Services, Edmonton, AB, Canada; ^7^Assistive Technology Laboratory, Faculty of Rehabilitation Medicine, University of Alberta, Edmonton, AB, Canada

**Keywords:** brain-computer interface, pediatrics, developmental neuroscience, brain-machine interface, clinical neuroscience, neurology, cerebral palsy

## Abstract

Thousands of youth suffering from acquired brain injury or other early-life neurological disease live, mature, and learn with only limited communication and interaction with their world. Such cognitively capable children are ideal candidates for brain-computer interfaces (BCI). While BCI systems are rapidly evolving, a fundamental gap exists between technological innovators and the patients and families who stand to benefit. Forays into translating BCI systems to children in recent years have revealed that kids can learn to operate simple BCI with proficiency akin to adults. BCI could bring significant boons to the lives of many children with severe physical impairment, supporting their complex physical and social needs. However, children have been neglected in BCI research and a collaborative BCI research community is required to unite and push pediatric BCI development forward. To this end, the pediatric BCI Canada collaborative network (BCI-CAN) was formed, under a unified goal to cooperatively drive forward pediatric BCI innovation and impact. This article reflects on the topics and discussions raised in the foundational BCI-CAN meeting held in Toronto, ON, Canada in November 2019 and suggests the next steps required to see BCI impact the lives of children with severe neurological disease and their families.

## Introduction

Pediatric brain-computer interface (BCI) is a rapidly developing subfield of BCI research, with its unique challenges, barriers, and advantages (Mikoajewska and Mikoajewski, 2014; Alves et al., 2016; Chau and Fairley, 2016; Kinney-Lang et al., 2016; Norton et al., 2018; Zhang et al., 2019). As awareness of the technology continues to grow in both research and clinical spheres (Kinney-Lang et al., 2016; Letourneau et al., 2020), so does the need for connecting pediatric BCI expertise. Building up cooperation between researchers, clinicians and stakeholders will be critical in the coming years to raise pediatric BCI to its full potential.

To this end, the initial BCI-CAN meeting brought together a bulk of the world’s leading experts in pediatric BCI alongside associated stakeholders, including families, advocates, and hospital foundation representatives, to the Holland Bloorview Kids Rehabilitation Hospital on November 15, 2019. The meeting focused on accomplishing a collective mission: establishing a formal, inclusive partnership across pediatric BCI experts and stakeholders to create a Canada-wide framework for advancing pediatric BCI to realize new opportunities for children with severe neurological disabilities.

Presentations from experts, sponsors, and families provided a holistic overview and insights into current pediatric BCI practices, goals, and challenges across the network. Paired with presentations were opportunities for both large group discussions and smaller group break-out sessions. This collaboration marks the first-of-its-kind for pediatric BCI research, engaging stakeholders across the spectrum to combine resources and spur innovation streams. The remainder of this article aims to summarize the topics discussed at the BCI-CAN meeting and directions for progress.

## Overview of Existing Pediatric BCI Programs in Canada

### Holland Bloorview’s PRISM Lab

The BCI program at Holland Bloorview is motivated by the unmet, pining need for communication and environmental interaction among many children and youth who present without expressive communication *via* speech or functional movements [e.g., individuals with cerebral palsy (CP) at Gross Motor Function Classification System (GMFCS) level 5; severe neuromuscular conditions such as congenital myopathies; childhood stroke; demyelinating leukodystrophies]. We have seen over the last decade an increasing number and diversity of children and youth for whom the search for a single motoric pathway of control is intractably elusive. The BCI program arose to fill this gap and complements on-going efforts to devise novel somatic controls (e.g., smile switch, dysarthric speech decoding, non-contact tongue switch, mechanomyographic switch).

Our BCI research deploys three modalities of brain interrogation, near-infrared spectroscopy (NIRS), transcranial Doppler ultrasound (tCD), and electroencephalography (EEG). The majority of our early work focused on the hemodynamic modalities (NIRS and tCD), where we demonstrated viable brain-based control using a variety of different communication interfaces with a broad range of mental tasks and self-regulation approaches, with both young adults and children. With improvements in EEG instrumentation, we have been investing more energy into EEG BCIs and NIRS-EEG hybrids. In addition to the usual evoked potential; e.g., P300 oddball response, Steady-State Visual Evoked Potentials (SSVEPs), or Auditory Steady-State Responses and motor imagery-based BCIs, one of our most promising developments include an auditory-tactile BCI that can allow children with visual impairments to use a BCI. With concomitant motor impairments, these children currently have very few options for communication (e.g., painstakingly slow and unforgiving auditory scanning). Some of our current efforts include the development of more intuitive communication interfaces (e.g., using human factor principles and methods), mental speech BCIs, and training protocols that accelerate the acquisition of BCI control.

Our research program is closely integrated with our BCI clinic, which was founded in 2019. Our BCI clinic team comprises clinical expertise in assistive technology, Therapeutic Recreation (TR), and biomedical engineering. The program is driven to make BCIs accessible to children with disabilities, a population often excluded from BCI research. Children with restricted control over movements and who face challenges finding a reliable assistive device for communication and environmental control are targeted to participate in this program. The main goal of the program is to provide access to BCI for recreation. Toys and games are ideal first activities when learning how to use BCI as it provides a motivating and engaging activity to master a mental task.

Our BCI clinic is divided into two streams: training and recreation. The training stream is where our participants first begin with BCI. They learn what BCI is, practice performing mental tasks, and explore a wide variety of toys and video games. Once the child demonstrates consistent control of the BCI over multiple sessions, they enter the recreation stream. The recreation stream allows the child to have continued access to BCI activities and provides the opportunity for experienced BCI users to engage in group BCI activities such as playing multi-player video games.

The two BCI clinic streams are extremely interconnected with each other as well as with the research program. Advancements in signal processing and machine learning elucidated in research are applied to the BCI clinic. Practical experience in the BCI clinic informs the development of research projects. Children move freely between the recreation stream and training stream as needed and engage in research projects aimed to improve their BCI performance.

### Alberta Children’s Hospital—A Clinical BCI Program

A clinical pediatric BCI program was founded at the Alberta Children’s Hospital (ACH) in 2017. The mission was to provide children with severe neurological disabilities and their families the opportunity to access and inform the development of practical BCI systems that could enhance their independence and quality of life.

The roots of the program lay in existing, complementary clinical research programs, specifically successful neurotechnology applications in CP populations including non-invasive neuromodulation clinical trials. This familiarity with clinical populations and applied neurotechnology combined with the existing highly skilled personnel including experts in neurology, rehabilitation, neuroscience, and biomedical engineering led to establishing a foundational pediatric BCI program.

In 3 years, the program has achieved multiple early aims. A recruitment program was developed that has connected 10–12 individuals and their families with the team and program, each with unique needs and goals. These participants encompass a broad range of clinical diagnosis but share an underlying phenotype of severe physical impairment (e.g., GMFCS level 5), minimal hand function (MACS level 5), either non-verbal or significant communication impairment, and evidence of at least a grade 1 cognitive capacity as assessed by parents, caregivers and clinicians. Initial recruitment has largely included participants with quadriplegic CP resulting from a variety of causes (perinatal stroke, hypoxic-ischemic injury, prematurity, brain malformations, and genetic/metabolic conditions) with other children and families fitting the general inclusion criteria also recruited. Multiple BCI technologies have been acquired and tested, ranging from simple EEG-based headsets to advanced control, communication, and rehabilitation systems.

Informed by the patients and families themselves, a growing number of applications have been developed, ranging from video games and recreational activities to communication systems, computer control, and art programs.

With a patient-centered focus, the program is ready to integrate with leading partners from industry, biomedical engineering, rehabilitation, and broader clinical centers to better realize the potential of BCI to improve the lives of children with a severe disability.

### Glenrose Rehabilitation Hospital—A Burgeoning Pediatric BCI Lab

A clinical pediatric BCI program was founded at the Glenrose Rehabilitation Hospital (GRH) as part of the I CAN Centre for Assistive Technology in spring 2019. The mission, aligned with provincial partners at the ACH Pediatric BCI Program, is to provide an opportunity for individuals with a severe neurological disability to access and inform the development of non-invasive BCI technologies to achieve greater independence and quality of life. The initial aim is to provide access to BCI as a “just try it” opportunity for children, as well as the exploration of BCI as an alternative technology access method to help meet goals around the use of technology for play/leisure, environmental control, communication, and mobility.

The BCI program at the Glenrose has at its core an existing foundation in cutting edge rehabilitation and assistive technologies, prioritizing patient and family engagement in all stages of development. The Glenrose I CAN Centre and Glenrose Rehabilitation Research Innovation and Technology (GRRIT) program has a long history of successful clinical integration of technology tools, as well as highly skilled experts in rehabilitation, assistive technology, engineering, and robotics.

Patient-centered translational research is a core element of the GRH BCI program vision, building on Glenrose’s strength of existing multidisciplinary academic partnerships from the Faculties of Rehabilitation Medicine, Engineering, and Medicine and Dentistry at the University of Alberta.

Our program’s initial phases have been focused on the acquisition and integration of BCI technologies, obtaining pilot funding, and establishing collaborative relationships with our provincial partners in Calgary (ACH), and participating in the establishment of the BCI-CAN national network. Our initial clinical focus has been to work with children and their parents to build skills, inform quality improvement, and to develop a BCI activity library that includes tasks that are engaging, motivating, and fun. The GRH team has been actively working to recruit and support a variety of clinical populations who could benefit from BCI ranging from children with quadriplegic CP (GMFCS levels 4 and 5) and childhood stroke to Rett Syndrome, posterior fossa syndrome, and traumatic brain injury.

## Technology and Research

### Pediatric BCI Technology: A Promising Outlook for the Future of Childhood Disability

The future of BCI technology is promising. Pediatric BCI technology in particular is seeing a surge of interest, with possible improvements in therapeutic outcomes already reported for various patient populations. Early research has suggested that through the assistance of a BCI, neurofeedback based rehabilitation in children with CP may help encourage early neuro-plastic changes which could improve the acquisition of motor control, which would otherwise be more challenging (Daly et al., 2014). As this field moves forward, collectively we should strive to improve the quality of life for both those with a documented history of motor control, as well as for those whose movement patterns and motor control may not have been previously apparent.

Varied future applications for BCI technology are on the horizon. These include a range of projects from improving the information transfer rate of speech and communication-based BCIs to evolving current pediatric BCI systems to include multi-modal data and input streams. These goals aim to meet the complex needs of individuals who will benefit the most from these systems while improving their autonomous communication, social responsiveness, and movement fluidity. Events like the Cybathlon are providing unique, competitive environments that help promote longer BCI use and practice with through BCI end-user (Perdikis et al., 2018). BCI teams across the world are invested in transforming lives which might benefit from the development and synthesis of BCI yet almost none are focused on the needs of disabled children.

The development of pediatric BCI systems faces its own set of unique challenges. BCIs depend on controllable, evoked patterns of brain activity for analysis. Due to the current dearth of pediatric BCI research, however, evoked activity in the developing brains of children has yet to be well characterized for BCI. Many existing BCI paradigms involve dry, repetitive tasks that do not take into consideration the shorter attention spans of children, prompting the need for more engaging BCI paradigms. Children are also likely to be more sensitive to fatigue and discomfort from wearing BCI hardware for long periods. Improvements in BCI hardware, signal processing methods, and classification algorithms can all contribute to reducing the overall amount of data that need to be collected, thus reducing the likelihood of fatigue and discomfort (Kinney-Lang et al., 2016). The underlying conditions will certainly differ in children (CP rather than ALS) where unique brain injuries, neurophysiology, and the effects of ongoing development must all be considered. Perhaps most importantly, the needs and goals of individual users will be different in children and youth, and patient-driven development of BCI technologies and applications is essential. These challenges, while prevalent and clear limitations for modern BCI systems should not be seen as insurmountable. Rather, they should serve as areas of focus for those motivated to advance BCI applications for children, with the understanding that overcoming these existing barriers will require extensive collaboration among BCI stakeholders across the spectrum.

### Hybrid Brain-Computer Interfaces

If BCIs are to be accepted clinically, it is critically important that the system can accurately predict user intent (Wolpaw, 2007; Daly and Wolpaw, 2008; Wolpaw and Wolpaw, 2012). Due to current hardware and software limitations, the classification accuracy of predictions is less than 100%. Even for typically developing individuals, accuracy ranges from 50 to 98 (Lee and Choi, 2018; Wang et al., 2018) and drops further for individuals with disabilities (Daly et al., 2014; Lazarou et al., 2018). Studies with children are few with variable results, some reporting lower performance (Mikoajewska and Mikoajewski, 2014; Kinney-Lang et al., 2016) while others suggest comparable results to adults (Zhang et al., 2019). Hybrid-BCIs may be one approach to help improve BCI accuracy.

Hybrid-BCIs combine different types of brain signals with other physiological measures or another access method to improve classification accuracy by increasing the confidence in the predictions made by the system (Wolpaw and Wolpaw, 2012; Choi et al., 2017). Researchers at Holland Bloorview Kids Rehabilitation Hospital have already begun research in this area. For example, classification accuracy improved in a covert speech task using a hybrid functional near-infrared spectroscopy (fNIRS) and EEG-BCI rather than using either alone (Rezazadeh Sereshkeh et al., 2019). Similarly, classification accuracy improved in a verbal fluency task using a hybrid fNIRS and transcranial Doppler ultrasonography (TCD) BCI rather than either alone (Faress and Chau, 2013). Also, researchers have looked at readiness potentials as a cue for identifying when movements are voluntary rather than unintentional, such as those seen in persons with athetoid CP. These techniques could be used to determine if a switch selection was accidental or intentional (Zeid et al., 2017). Another area to be explored is monitoring the brain’s error-related potential, which occurs when a user knowingly observes an erroneous selection occurring, permitting a BCI system to self-regulate when a prediction was incorrect (Schalk et al., 2000). Techniques such as these appear to be applicable in youth and might be expanded and trialed across a pediatric BCI network.

In addition to improving classification accuracy, hybrid BCIs can extend the control capacity of BCI systems. Hybrid BCIs can be used to initiate asynchronous control, with one of the systems used to activate the other. This is highlighted in Pfurtscheller et al. (2010) “brain switch” device, where motor imagery was used to turn on flashing LED lights for an SSVEP—controlled hand orthosis. Similarly, hybrid BCIs can be used to separate target identification and target selection, with one system used to guide the user towards the desired target and the other to select the target, such as in combined eye-gaze and EEG BCIs (Kim et al., 2015). Hybrid BCIs can also be used to increase the dimensionality of target selection, combining motor imagery with visual evoked potentials to achieve two-dimensional cursor control (Ma et al., 2017). Whether improving classification accuracy or increasing functionality, hybrid BCIs facilitate the utilization of the strengths of different BCI modalities while mitigating their limitations. This approach may be fruitful in designing BCI systems that address the unique needs of pediatric users.

### Tapping Into Play: Games and Play in BCI

Play is a critical part of learning and development for all children. Both neurotypical and neurodivergent children benefit more from participating in tasks that keep them interested, engaged, and provide embedded learning opportunities. Current BCI software, however, tends to focus on simple, utility-driven applications, such as spelling grids or moving a mouse cursor. While useful, such applications are limited in an appeal for sustained use, particularly for young BCI-users. Evidence indicates that increasing engagement in BCI through gamified learning may result in longer adoption of the technology while helping promote the practice of BCI control schemes (Powers et al., 2015; De Oliveira et al., 2016; Kinney-Lang et al., 2016; Mullins, 2017). A growing trend across BCI research endeavors reveal that more engaging, user-friendly activities may be able to promote a variety of tangible boons in BCI use—both in short-term task learning and long-term BCI accuracy (Perdikis et al., 2018; Edelman et al., 2019; Faller et al., 2019). Thus, there is a clear need to promote developing more captivating, accessible BCI software which incorporates essential elements of play into pediatric BCI.

BCI systems offer unique and attractive opportunities for novel approaches to both virtual plays (e.g., videogames and digital mediums) and physical play (e.g., manipulation of toy robots, cars, et cetera). By tapping into the non-muscular nature of BCI, such systems may be able to provide previously excluded populations the opportunity to explore and learn through play. Previous research has demonstrated such mediums as important avenues that encourage continued learning and rehabilitation in children with disabilities (Harris and Reid, 2005; Howcroft et al., 2012; Hernandez et al., 2013; van den Heuvel et al., 2016). Advancements in BCI research furthering the interaction between BCI systems and play thus represent a promising untapped potential for pediatric BCI end-users.

### Current Technological and Practical Limitations of Modern BCIs

Despite its significant potential, modern BCI systems and applications suffer from several limitations, which are only compounded when implementing programs with pediatric populations (Mikoajewska and Mikoajewski, 2014; Kinney-Lang et al., 2016; Letourneau et al., 2020). Such barriers exist across the BCI experience: from technological limitations, through design issues affecting user-experience, to long-term implementation challenges (Millán et al., 2010; Powers et al., 2015; Lazarou et al., 2018; Lotte et al., 2018). Highlighting some of these existing limitations may help shed light on potential pathways to investigate. At the technological level, currently, available BCI systems can be limited by their trade-off between accuracy, speed, and degrees of freedom for selection. Moreover, these systems are designed without consideration for children as potential end-users, leading to preconfigured signal processing and analysis schemes which may neglect neurophysiological differences between adults and children (Cowan et al., 2006; Vuckovic et al., 2014; Kinney-Lang et al., 2018a, b), At the user experience level, BCI may be limited in the comfort of headsets, slow “set-up” times, as well as difficulties in maintaining attention and control for extended periods due to fatigability. Finally, at the implementation level, relevant applications of interest may be limited for end-users, particularly children, as few long-term engagement applications have been explored with BCI systems leading to a potential drop in motivation and an increase in frustration as time is invested in learning the BCI control system.

## Clinical Systems and Targets

There is a paucity of literature regarding the use of BCI in children with complex motor and sensory issues (Kinney-Lang et al., 2016). BCI exploration, trial, and intervention in this national network, with interdisciplinary communication and collaboration, may be able to overcome this. This in turn may facilitate guideline development (Brumberg et al., 2018), with procedures to introduce BCI as an alternate access option across a broad clinical spectrum. Early clinical implementation will allow software and hardware features to be identified that contribute to successful implementation as well as better identify which children may benefit most from BCI technology access through clinical case studies and generation of research questions. The clinical implementation also allows clinicians to provide valuable feedback to researchers about practical issues that children with complex neurological and motor challenges face that may not be captured in trials with typically developing children. Clinicians will be able to share and provide strategies around areas such as positioning, tolerability of headsets/electrodes, timing issues, and ways to communicate/engage with this client population. Most importantly, a patient- and family-centered approach will ensure the needs of those most affected are informing the research questions and driving progress in relevant directions.

### Patients and Families First: Engagement to Inform Aims and Progress

Patient-centered approaches are essential to ensure the translation of research efforts into a meaningful benefit for affected children and their families. There are likely many benefits to this approach including recruiting interest in, and informing about, BCI capabilities for patients. These benefits in turn can inform more accurate and achievable goals driven by patients and families. A patient-centered frame of reference emphasizes the goal of independent participation in meaningful activities for the child and may help support increased engagement when using BCI technology as an access method. Looking at how patient-first perspectives at the ACH have helped shape the successful implementation of a pediatric BCI clinical program may help provide insights for other newly developing pediatric BCI collectives.

The ACH BCI program is relatively new and methods are evolving. Potential candidates are identified by surveying related clinicians and specialists (neurologists, physiatrists, occupational, physical, and speech therapists) for children with specific characteristics. To begin with, a relatively simple inclusion criterion was sought of a school-aged child with severe neuromotor impairment (GMFCS level V, no or minimal hand use), impaired communication (non-verbal or very limited), and high suspicion by family and/or clinicians of at least a grade 1 level cognitive capacity. Potential candidates are then further screened by the lead neurologist regarding underlying diagnosis, results of neuroimaging and electroencephalographic testing, and other variables that might affect their candidacy. As spaces become available, the top candidates are then invited to the neurology clinic for an assessment where a general introduction to BCI and the program is carefully explained before enrollment is offered.

The intake process uses concepts from The Canadian Model of Occupational Performance and Engagement (CMOP-E; Polatajko et al., 2007) and the SETT Framework (Student, Environment, Task, Tools; Zabala, 1995; Bryans-Bongey, 2018) to gather information about the child, their occupation (play and academia), their environment (home and school) and the current and past tasks and tools (assistive technology) used. A holistic gathering of the child’s functional level including physical, cognitive, sensory, academic goals, and preferred/non-preferred tasks is obtained from a medical file review, parent report, and community/school liaison. The intake information is used to set up the room environment and choose initial session activity choices to meet the needs of the child. The children participate in their typical mobility and/or positioning device that is used at home and school. Alternative positioning equipment is offered to the child if they desire to continue participating in an activity but would benefit from a more supportive position to perform the BCI task (visual imagery or attention to visual stimulus), promoting a positive outcome for the child.

The child’s key role and participation in the development of the BCI program are seen through their provided input and perspective in preparation of, and during, a session. Parent involvement is encouraged to assist their child’s participation. The patient-centered practice is implemented during both the initial session(s) and as homework. For example, both the child and parent are integral in helping direct the child’s “personalized” visual imagery task to be used in the introductory BCI session. In this example, the homework aspect would require the child and parent to explore, and consequently practice, one of the agreed-upon imagery paradigms (e.g., imagined actions) at home. This practice may take the form of repeatedly imagining the designated action without a typical research-grade BCI system providing feedback. Anecdotally, such homework encourages the consistent practice of designated motor imagery and aims to improve familiarity with the BCI-control requirements. Ongoing efforts are underway to provide commercial-grade systems to families to facilitate such potential homework activities. It is important to note that such at-home systems are considered as supplemental tools to facilitate engagement with BCI for the pediatric population, not necessarily to replace existing at-home assistive technology solutions. The many challenges of transitioning BCI systems to the home that have been shown in adult populations may also require special consideration for children.

As successful visual imagery can be different for everyone, working with the families and children to choose an effective scheme is of high priority. Input from participants is also used to determine their preference, enjoyment, and views on trialed activities as well as the level of difficulty perceived and other challenges during a session. Feedback is incorporated and discussed with the families and children to determine if they would like to perform such activities again, reset their goals, or try something new.

The importance of a patient-first practice cannot be understated. The children who have participated in the above program have reported strong feelings of self-identification and a sense that they are a part of the “research team” for developing pediatric BCI technology. These responses magnify their level of engagement in the program. How all of the above elements are accurately measured and tracked over time to evaluate outcomes and quality improvement are currently being studied.

### Establishing Ground Truth: Baseline Performance and Outcome Measures

As an emerging field, we must first establish how well typically developing children can operate BCI systems. A primary goal of the network is to evaluate baseline levels of performance across leading EEG-BCI systems and paradigms that are well-established in adults. Baseline measures to consider include classification accuracy of established systems, as well as scores for the agreement between the requested task and the performed task by the children, often measured by Cohen’s kappa. The evaluation of user experience is also essential including measures of tolerability and motivation—factors that are likely to affect performance. Studies are required to determine how to best improve the measurement of these factors, thereby advancing pediatric BCI performance and contributing to the advancement of our clinical pediatric BCI program for children with disabilities.

Outcome measures from a clinical BCI program should be first and foremost patient- and family-oriented. Our vision is to bridge the gap between emerging BCI technologies and the patients and families who stand to benefit from their use by creating an environment that engages patients and families to inform and drive technological progress. Primary outcomes in this environment are defined as subjectively meaningful goal achievement which may include improvement in functional abilities, independence, or otherwise enhanced quality of life for the child and/or family. Measurements from these outcomes rely on continuous active engagement from both patients and families in which they can relay their needs, goals, perceived successes, and failures, etc., from the program. Validated approaches in other forms of childhood neurorehabilitation include tools such as the Canadian Occupational Performance Measure (COPM) and goal attainment scaling (Kiresuk and Sherman, 1968; Law et al., 1990; Polatajko et al., 2007). Acquiring such subjective outcome measures might be facilitated through the use of regular semi-structured interviews in addition to the development of a BCI-support group. Secondary outcomes may focus on the technological aspects of improving the BCI technology for pediatric use, through concepts like personalization and optimization of the BCI schemes for unique participants. Within these secondary outcomes, priority should focus on developing well-defined, validated screening programs to assess and gauge the suitability of various BCI paradigms and control systems for potential candidates, customizable home-based BCI solutions for the realization of individual goals, and integration with other assistive technologies to create new opportunities for individual success and functional use.

### Augmentative and Alternative Communication

Clinical BCI programs can provide access to innovative non-invasive BCI technologies that might allow children to participate in meaningful communication activities for greater well-being and improved quality of life. Brumberg et al. (2018) highlight the importance of interdisciplinary collaboration along with clinical research to adapt protocols to support BCI intervention. A pan-Canadian clinical collaboration allows clinicians to consider BCI as alternate access means to address assistive technology and augmentative and alternative communication (AAC) goals. The Augmentative Communication and Educational Technology Service (ACETS) in Calgary, the I CAN Centre for Assistive Technology (ICAN) in Edmonton, and PRISM lab at Holland Bloorview in Toronto represent teams which support excellence in communication and assistive technology for meaningful living. Team missions are aligned across sites, promoting evidence-informed practice and collaboration to support opportunities for children and youth living with severe neurological disabilities. Through a “just try it” approach, BCI can be accessible to children and youth with severe motor and sensory impairments.

The centers use a variety of commercially available systems with communication capacities ranging from very basic headsets such as the Emotiv EPOC (Nijboer et al., 2015) to more sophisticated BCI systems. Each can be modified and paired with customized software to create personalized BCI solutions with multiple possible communication applications. New equipment and software applications are evolving rapidly and can be tested and evaluated across centers to explore alternative and innovative approaches. Individual children’s needs and preferences can inform and generate an inventory of equipment and technology solutions to meet these varied needs. It is essential that BCI programs engage with, and recruit the expertise of, colleagues in AAC whose clinical and technological experience is invaluable in defining and developing patient-specific solutions.

### Recreational Therapy

TR provides experiences of meaningful engagement, empowerment, and identity formation (Carruthers and Hood, 2007; Hood and Carruthers, 2007). This in turn can impact the subjective experience of well-being, in the context of leisure pursuits, and brings with it the opportunity to learn and develop skills (Carruthers and Hood, 2007; Hood and Carruthers, 2007). BCI facilitation can support the fruition of these outcomes for clients who require adaptation and enhanced activity engagement. A collaborative TR/BCI technology approach has the potential to enhance functional capacity, connectivity, and provide meaningful leisure engagement that might strengthen health, wellness, and quality of life for both children and adults as well as their families and social networks. Across our fledgling network, there are already many highly meaningful examples including kids playing video games, controlling social media, driving remote control cars, and creating original works of art; all things they had never previously been able to do themselves.

Independently engaging in leisure for the first time can be life-changing for a child with severe neurological impairment. BCI technology creates ability and possibility, opening up legions of new activity prospects. In the context of leisure, BCI creates opportunities for independent control of games, toys, and activities that would otherwise require hand-over-hand or partner assistance. Through BCI, freedom in choice and action are reclaimed by individuals who may have lost, or never had such capabilities. We cannot underestimate the impact such independence and choice can have on an individual’s ability to flourish in leisure and life.

## Building The Collaborative BCI-CAN Network: Group Discussions

### How to Synchronize Technical Advancements Across the BCI-CAN

To create a unified coalition across pediatric research sites, it was deemed important to outline goals that guide our teams concerning technological advancements and data sharing. A primary aim was to identify which mechanisms could be leveraged to push research forward at each site quickly while maintaining enough similarity to promote easy sharing of resources, methods, and knowledge across sites. Participants discussed establishing common BCI hardware at each site to facilitate multi-site studies and increase the number of clients that could be engaged in a single research study with standardized methods. Also, it was proposed to create a multisite ethics application to reduce the amount of time spent on gaining ethics board approvals for multi-site studies. Through seeking multi-site ethics approval, the collaboration will reduce the amount of time spent on individual research ethics applications for proposed collaborative studies. Developing data and code repositories were also considered to encourage sites to build upon each other’s progress, promoting open science, reproducibility, and large datasets amenable to advanced analytic techniques. Finally, establishing effective communication channels for cross-site updates was deemed important to strengthen the coalition and encourage collaboration.

Of particular interest was the topic of which technology (hardware/software/approaches) would need to be common across sites and which BCI technology might be more flexible. Participants agreed it was imperative to match the quality of signal acquisition for BCI across sites, but not necessarily exact amplifiers or BCI brand names. For example, if each site has a medical-grade EEG system, but these were produced by different manufacturers, the quality should remain comparable enough for multi-site data collection. Consistent data quality would allow the network to share BCI algorithms and progress their development and evaluation. Legal agreements would need to be put in place before sharing such codes and data but were not considered a significant barrier. The development of a representative coalition was proposed to institute channels for communication and sharing of information across the network.

### Promoting the Next Generation of Pediatric BCI Researchers

A trainee “pre-conference” meeting was held to generate discussion and gain insights into the key topics important to trainees and early-career researchers interested in pursuing pediatric BCI technology and applications. The trainees developed their thoughts through a series of brainstorming sessions in the form of small-group discussions on designated topics. This environment provided space for these researchers to contribute suggestions for realizing the multi-site collaboration and to express their opinions on key related topics. The most prevalent suggestions are summarized in Table 1.

**Table 1:** Suggestions from the group discussion on formalizing support for the BCI-CAN network.

	Communication across network	Cross-site Leadership	Cross-site Collaboration	Participation in BCI Events
**Primary goal:**	Facilitate continued communication across teams	Facilitate trainee learning and professional development	Develop opportunities for trainees to contribute to collaborative research	Encourage trainees to attend, host and discuss BCI-related events
**Tangible Outcome**	Arrange a monthly virtual meeting across the sites	Establish teaching and learning opportunities for all trainees	Develop pathways required for multi-site collaboration	Build collaboration across the network through BCI events
**Key Considerations**	Organized by trainees and research staff	Develop an on-boarding BCI crash course	Encourage trainee participation in multi-site studies	Organize and/or compete in BCI-related competitions
	Opportunity to present trainee ideas and updates	Provide an opportunity for mentoring and mentorship	Establish an internship program for trainees to visit, learn and teach at each site	Present alongside other trainees at BCI based conferences
	Allow time for support and feedback requests	Share available resources and courses	Implement resource sharing among trainees for data, codes, protocols, etc.	Organize and attend BCI specific workshops and seminars

One important outcome in supporting the next generation of trainees is to promote standardization in scientific practice and BCI implementation across all network members. To promote such scientific unity, senior trainees put together a series of online workshops from early April to June 2020 focused on foundational topics in BCI. These workshops were developed with the primary goal of facilitating trainee learning across the network (Table 1; column 2) during the COVID-19 pandemic. The topics included a general introduction to BCI, an in-depth dive into currently available hardware including systems currently used at each center, and a technical introduction to processing EEG signals. Additional workshops were held to provide trainees an essential understanding of the MATLAB and Python coding languages. These workshops will be repeated annually, with additional workshops added if they can effectively support skill development and standardization across the network.

### Growing the Network: Opportunities and Pathways to Build Better Pediatric BCI

Each current site affiliated within the BCI-CAN network has unique strengths to contribute to the collaborative network. Holland Bloorview’s research in furthering BCI from a biomedical engineering perspective is providing key developments in various signal acquisition and processing modalities and is now complemented by a parallel clinical program. ACH’s clinical orientation is emphasizing patient-centered care and research within an environment experienced in applying advanced neurotechnologies in experimental clinical trials. Glenrose’s focus on the integration of BCI within their wide breadth of clinical assistive technologies for AAC is leading to new implementation schemes for less experienced BCI users and teams. To grow as a network, we will utilize and expand these respective existing strengths to support individual and collaborative projects spanning across the network. To support collaborations and growth, monthly meetings are held with attendance from the network as a whole. Also, more niche communications within and between each program are held to discuss ways to best support collective goals at the sites. Such meetings can include engineering teams (e.g., members focused on BCI software and hardware development) or clinical teams (e.g., members focused on user experience and BCI implementation). The primary goal for the collective network over the coming years is the establishment of structured BCI programs at each site with clear pathways for support across sites.

Another unique potential advantage for our network is the opportunity for exchanges, knowledge translation and recruitment between the sites, and future expansion. Affiliation with the network will be beneficial for research sites by providing interdisciplinary training opportunities, shared resources, and facilitated growth of trainees in various aspects of pediatric BCI research. While the original members in the network have been Canadian-based (with new national centers expressing interest), we have recently engaged other North American groups, such as the NCAN Centre (New York, NY, USA), and envision growing as an international coalition of pediatric BCI groups across the globe. To join our network, please contact the corresponding author (EKL).

## Discussion

The first BCI-CAN network meeting concluded with a clearer vision of our collective short-term aims: (1) to organize efficient communication schemes that promote continued conversations, collaborations, and relationships across the network; (2) to establish a trainee and research team network that can promote learning experiences, knowledge and data sharing, career development, and the education of all stakeholders; and (3) to collectively pursue opportunities for network growth *via* both national and international collaborations.

Since this foundational meeting, we have had consistent, positive engagement from across the network. We have hosted over a dozen meetings between the sites, organized multiple learning opportunities on topics ranging from an introduction to core concepts in pediatric BCI to introductory programming basics in MATLAB and Python. We have initiated a multi-site study across our network to evaluate and develop BCI-driven video games for children with severe neurological disabilities which was presented at the international IEEE Engineering in Medicine and Biology Conference 2020 (Kelly et al., 2020; Kinney-Lang et al., 2020). Additionally, we have collaboratively organized pediatric-focused BCI workshops for international meetings (8th International BCI Meeting, Brussels, Belgium) and recently established a seminar series with guest speakers from other BCI groups across the globe. Upcoming activities include both in-person and virtual conference meetings rotated across sites, cross-center technical development of novel BCI technologies with testing with participants in different cities, patient and family engagement across centers including online social networking, and many more in pursuit of our primary aim of helping severely disabled youth realize new opportunities with BCI.

## Ethics Statement

**Figure 1:**
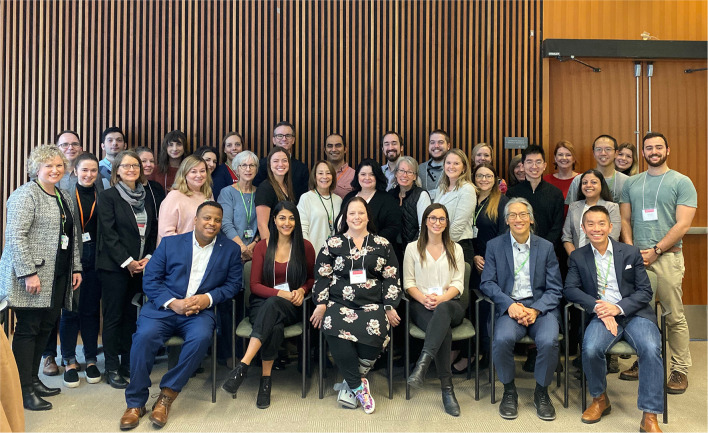
Photo of participants from the first annual brain-computer interfaces (BCI) Kids Canada Meeting held on Nov. 15th at Holland Bloorview Kids Rehabilitation Hospital, Toronto, ON, Canada.

Written informed consent was obtained from individuals in Figure 1 for the publication of any potentially identifiable images or data included in this article.

## Author Contributions

This work represents a collective contribution and summary from all authors on this manuscript. EKL collated the accounts and descriptions together, with supervision from AK. All authors contributed to the article and approved the submitted version.

## Conflict of Interest

The authors declare that the research was conducted in the absence of any commercial or financial relationships that could be construed as a potential conflict of interest.
